# Can Night Shift Work Affect Biological Age? Hints from a Cross-Sectional Study on Hospital Female Nurses

**DOI:** 10.3390/ijerph182010639

**Published:** 2021-10-11

**Authors:** Michele Carugno, Cristina Maggioni, Vincenzo Ruggiero, Eleonora Crespi, Paola Monti, Luca Ferrari, Angela Cecilia Pesatori

**Affiliations:** 1 Department of Clinical Sciences and Community Health, Università degli Studi di Milano, Via San Barnaba, 8, IT-20122 Milan, Italy; cristinamaggioni.md@gmail.com (C.M.); luca.ferrari@unimi.it (L.F.); angela.pesatori@unimi.it (A.C.P.); 2 Occupational Health Unit, Fondazione IRCCS Ca’ Granda Ospedale Maggiore Policlinico, Via San Barnaba, 8, IT-20122 Milan, Italy; paola.monti@policlinico.mi.it; 3 Faculty of Medicine, Università degli Studi di Milano, Via Festa del Perdono, 7, IT-20122 Milan, Italy; vincenzo.ruggiero@studenti.unimi.it; 4 Occupational Health Unit, ASST Santi Paolo e Carlo, Via Antonio di Rudinì, 8, IT-20142 Milan, Italy; eleonora.crespi@asst-santipaolocarlo.it

**Keywords:** working schedule, epigenetics, biological aging, DNA methylation, overweight, obesity, work-related stress

## Abstract

Disruption of age-related processes seems to play a relevant role in health effects related to night shift (NS) work. We aim to verify whether NS work can influence biological age (BA), estimated through Zbieć-Piekarska’s epigenetic signature, based on methylation of five CpG sites in *ELOVL2*, *C1orf132*/*MIR29B2C*, *TRIM59*, *KLF14,* and *FHL2*. Forty-six female nurses working in NS were matched by age and length of employment with 51 female colleagues not working in NS. Each subject filled in a questionnaire (including the Effort Reward Imbalance (ERI) index to assess job stress) and gave a blood sample. Age acceleration (AA) was estimated by regressing BA on chronological age and taking the residuals. Multivariate linear regression models were applied. BA was not associated with NS. However, we did observe an increase in AA per each year in NS in subjects with overweight/obesity (β = 0.46, 95% CI: 0.05; 0.87, *p* = 0.03), experiencing work-related stress (β = 0.58, 95% CI: 0.10; 1.06, *p* = 0.018), or both (β = 0.66, 95% CI: 0.03; 1.29, *p* = 0.041). Although based on a small sample size, our findings suggest an increased BA only among hypersusceptible subjects and is worth further investigation, also in light of recent results suggesting a higher breast cancer risk in women with increased AA.

## 1. Introduction

Shift work, especially one involving night shifts (NS), has been associated with an increased risk of mortality and a higher incidence of cardiovascular diseases and cancer [1,2,3]. Chronological age is a well-known risk factor for chronic diseases, mortality, and functional impairments. However, there is still great heterogeneity in the health outcomes of older individuals, making the search for markers that track the state of biological aging very relevant. Shift work involves a variety of physiological disruptions [4] that might entail alterations in shift workers’ biological aging. Preliminary findings have underlined a possible relationship between shift work and aging by showing an association between duration of NS work and reduced telomere length [5,6], although its use as a potential index of biological aging is still controversial [7]. Other studies observed a high correlation between the methylation status of multiple candidate chromosomal loci and biological age [8], thus considering DNA methylation estimated age as a suitable predictor of biological age. Different methylome-based methods to estimate biological age have been proposed (Horvath, Hannum, and Levin “epigenetic clocks”). The Horvath clock is based on DNA methylation from cells belonging to multiple tissues [9], while the Hannum clock is based on DNA methylation from blood cells only [10]. Both were designed to predict chronological age. On the other hand, the Levin clock was conceived to estimate “PhenoAge”, a previously developed biological age metric based on the combination of chronological age and selected blood parameters [11]. All these age estimators showed a high and concordant correlation with chronological age [12]. However, they are characterized by a high number of DNA methylation loci that need to be examined, which might be a limiting factor for their applicability in observational studies. A more parsimonious estimator was proposed by Zbieć-Piekarska and colleagues, which included five CpG sites whose methylation status explained 94% of age variance in their study population [13] and could thus be considered an epigenetic signature of biological age. We used this estimator to verify whether NS work can influence biological age.

## 2. Materials and Methods

### 2.1. Study Population, Personal Data, and Biological Samples

Data and blood samples were collected in a previous study [5] on 46 female nurses who had been working in NS for at least two years at the time of recruitment, matched by age and length of employment with 51 female colleagues not working night shifts (NNS). Participants were recruited on a voluntary basis among the female nurses of the Policlinico Hospital in Milan (Italy) while undergoing routine health surveillance. Information on demographics, personal and family health history, lifestyle, and work (with a particular focus on shift work schedule and duration) was collected through a semi-structured interview. To assess work-related stress, we used the Effort–Reward Imbalance (ERI) questionnaire [14], a standardized, self-reported measure of poor reciprocity between job efforts and job rewards which also takes into consideration overcommitment (a coping strategy for workers striving for approval) [15]. Work-related stress was defined as present with an ERI index > 1. After signing informed consent, each subject donated a 12 mL blood sample for DNA methylation analysis.

### 2.2. Analysis of DNA Methylation

Methods for DNA extraction and methylation analysis have been previously described [16]. Biological age was calculated considering the methylation pattern of 5 CpG sites in five genes (*ELOVL2*, *C1orf132*/*MIR29B2C*, *FHL2*, *KLF14*, *TRIM59*), according to the methodology proposed by Zbieć-Piekarska and colleagues [13]. The DNA samples (500 ng) were treated with sodium bisulfite using the EZ-96 DNA Methylation-Gold™ Kit (Zymo Research; Irvine, CA, USA). Bisulfite-treated template DNA was then amplified using the GoTaq Hot Start Green Master mix (Promega, Madison, WI, USA). Pyrosequencing was performed with the PyroMark MD System (QIAGEN GmbH, Hilden, Germany). Every sample was tested twice for each gene to guarantee the reproducibility of the experimental setting.

### 2.3. Statistical Analysis

Age acceleration was estimated by regressing biological age on chronological age and taking the residuals of the model (thus guaranteeing that age acceleration is independent of chronological age). The association between age acceleration and selected variables of interest was evaluated applying univariate and multivariate linear regression models adjusted for BMI (<25 vs. ≥25), ERI (≤1 vs. >1), and smoking habit (former/never vs. current). Night shift was considered both as current NS vs. NNS and as ever vs. never NS. We also considered “number of years worked in NS” as a variable of interest, which was equal to 0 in never night-shifters. The models assessing the association between the number of years in NS and age acceleration were run on the entire study population, also including the variable “never vs. ever NS” [17]. The interaction was assessed fitting potential effect modifiers in the models as multipliers. Results are expressed as regression coefficients (β) and corresponding 95% confidence intervals (95% CI).

## 3. Results

The main characteristics of the study population are reported in Table 1. The mean age was 35.9 (±5.4) years, and the mean duration of employment was 11.8 (±6.9) years. The distribution by BMI, smoking status, ERI, and oral contraceptive use did not differ between NS and NNS workers. The proportion of nulliparous was higher in NS nurses (89.1% vs. 56.9%, *p* = 0.002).

Chronological age was strongly correlated to biological age (r = 0.82, *p* < 0.001). In univariate regression models neither smoking (β = −0.95, 95% CI: −2.47; 0.57), nor BMI (β = −0.39, 95% CI: −2.00; 1.22), nor ERI (β = 1.57, 95% CI: −0.44; 3.59) influenced biological age.

**Figure 1:**
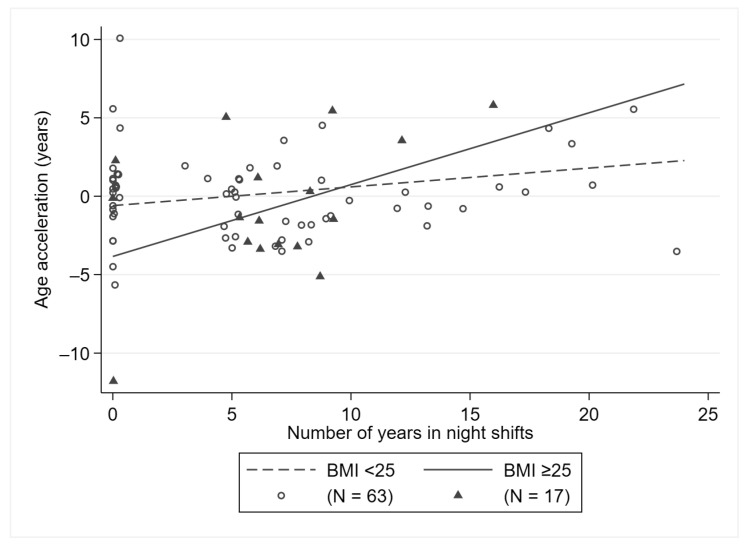
Association between the number of years in night shifts and age acceleration, stratified by BMI.

In multivariate models, biological aging was not associated with working in NS (current NS vs. NNS: β = −0.04, 95% CI: −1.4; 1.33; ever vs. never NS: β = −0.07, 95% CI: −1.57; 1.43) or number of years in NS (β = 0.14, 95% CI: −0.03; 0.32). Only subjects with overweight/obesity showed an increase in age acceleration per each year in NS (β = 0.46, 95% CI: 0.05; 0.87, *p* = 0.03, *p* for interaction = 0.097, Figure 1).

**Figure 2:**
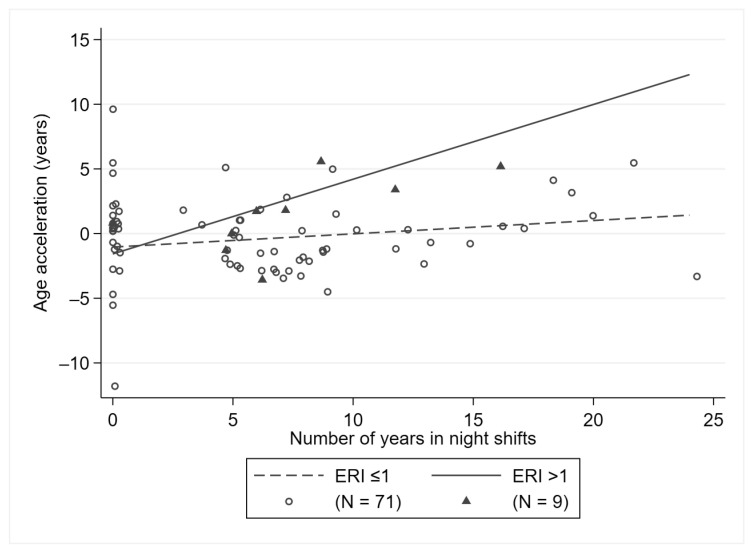
Association between the number of years in night shifts and age acceleration, stratified by ERI.

A similar pattern was observed for subjects experiencing work-related stress (ERI > 1), with an age acceleration of 0.58 years (95% CI: 0.10; 1.06, *p* = 0.018, *p* for interaction = 0.056) per each year in NS (Figure 2). We observed a higher age acceleration (β = 0.66, 95% CI: 0.03; 1.29, *p* = 0.041) when considering both categories combined (BMI ≥ 25 and ERI > 1), even if no formal interaction was apparent.

## 4. Discussion

We evaluated the association between biological aging and shift work, comparing nurses employed in night and day shifts.

The correlation we observed between chronological and biological age (r = 0.82, *p* < 0.001) is similar to the coefficients estimated by three methylome-based clocks [9,10,11].

In the whole population, smoking, BMI, and work-related stress were not significantly associated with biological aging. In addition, neither NS nor the number of years in NS appeared to influence biological aging. However, we did observe an increase in biological aging with the number of years in NS in subjects with BMI ≥ 25 or ERI > 1. Such age acceleration was even more prominent in the subgroup of women with both characteristics, with an increase of 0.66 years in biological age for each year working in NS. Our results thus suggest overweight/obesity and work-related stress as possible factors of hyper susceptibility to environmental (i.e., non-endogenous) stimuli. Indeed, both conditions have been identified as sources of low-grade systemic chronic inflammation, which can interact with other triggers related to shift work (such as disturbed sleep and disrupted circadian rhythms) in fostering the aging process [18,19].

To the best of our knowledge, only one study has so far examined biological aging in female shift workers [20], comparing Hannum’s, Horvath’s, and Levine’s epigenetic clocks between 175 female shift workers and 2399 women not employed in NS, aged 35–74 years. All three methods highlighted increased age acceleration per each year of overall or night-specific shift work, with the strongest association for ≥10 years of NS work. Our results are only partially consistent with this investigation, as we observed increased age acceleration associated with the number of years in NS only in a subgroup of women characterized by high BMI and/or high work-related stress. The differences we observed might relate to various factors, such as the small sample size and the younger age of our study population, as well as the different method we used to estimate biological age.

A previous investigation in the same population showed a decreased telomere length (another potential index of biological aging) in women employed in NS ≥ 12 years [5]. Telomere shortening after brief exposures to environmental *noxae* entails cell death and a continuous cell turnover that brings to an increase in new cells with long telomeres. The process of cell turnover only runs out after long exposures to environmental stimuli, and the pool of cells characterized by shortened telomeres eventually prevails. In the present study, we observed an increased age acceleration associated with the duration of NS only in subjects characterized by low-grade chronic inflammatory conditions. We can thus speculate that a higher level of inflammation is needed in order to determine a detectable change in DNA methylation.

## 5. Conclusions

Although based on a small number of subjects, our results suggest increased biological aging only among hypersusceptible subjects (affected by overweight/obesity and/or exposed to work-related stress). These findings are worthy of further investigation, also in light of the results by Kresovich et al. suggesting a higher breast cancer risk in women with increased age acceleration [12]. Moreover, should they be confirmed, they might also indicate hyper susceptibility conditions to be adequately surveilled in occupational settings.

## Figures and Tables

**Table 1:** Characteristics of the study population.

Characteristic		Current Night Shift		
*Mean ± SD, N (%)*	Total	No (51)	Yes (46)	p *
Age	35.9 ± 5.4	36.5 ± 5.3	35.3 ± 5.6	0.31
Duration of employment	11.8 ± 6.9	12.7 ± 7.3	10.7 ± 6.3	0.17
BMI				
<25	72 (74.2)	40 (78.4)	32 (69.6)	
≥25	21 (21.7)	10 (19.6)	11 (23.9)	0.52
Missing	4 (4.1)	1 (2.0)	3 (6.5)	
Smoking habit				
Former/Never	67 (69.1)	38 (74.5)	29 (63.0)	
Current	27 (27.8)	12 (23.5)	15 (32.6)	0.36
Missing	3 (3.1)	1 (2.0)	2 (4.4)	
ERI				
≤1	80 (82.5)	42 (82.3)	38 (82.6)	
>1	13 (13.4)	8 (15.7)	5 (10.9)	0.77
Missing	4 (4.1)	1 (2.0)	3 (6.5)	
Oral contraceptive use				
No	58 (59.8)	33 (64.7)	25 (54.4)	
Yes	34 (35.0)	16 (31.4)	18 (39.1)	0.36
Missing	5 (5.2)	2 (3.9)	3 (6.5)	
Number of children				
0	70 (72.1)	29 (56.9)	41 (89.1)	
1	12 (12.4)	10 (19.6)	2 (4.4)	
2+	15 (15.5)	12 (23.5)	3 (6.5)	0.002

SD: standard deviation. * Student’s *t*-test for continuous variables, chi-squared and Fisher’s exact tests for categorical variables.

## Data Availability

Data can be accessed upon reasonable request to the corresponding author (michele.carugno@unimi.it).
